# Oral delivery of a functional algal-expressed TGF-β mimic halts colitis in a murine DSS model

**DOI:** 10.1016/j.jbiotec.2021.08.006

**Published:** 2021-11-10

**Authors:** Danielle J. Smyth, Bijie Ren, Madeleine P.J. White, Caitlin McManus, Holly Webster, Vivien Shek, Caroline Evans, Jagroop Pandhal, Francis Fields, Rick M. Maizels, Stephen Mayfield

**Affiliations:** a Wellcome Centre for Integrative Parasitology, Institute of Infection, Immunity and Inflammation, University of Glasgow, UK; b California Center for Algae Biotechnology, Division of Biological Sciences, University of California, San Diego, USA; c Bioanalytical Facility, Dept Chemical and Biological Engineering, University of Sheffield, UK

**Keywords:** Algae, Helminth, Treg, TGM, TGF-β, Colitis

## Abstract

Inflammatory bowel disease (IBD) is a set of immunological disorders which can generate chronic pain and fatigue associated with the inflammatory symptoms. The treatment of IBD remains a significant hurdle with current therapies being only partially effective or having significant side effects, suggesting that new therapies that elicit different modes of action and delivery strategies are required. TGM1 is a TGF-β mimic that was discovered from the intestinal helminth parasite *Heligmosomoides polygyrus* and is thought to be produced by the parasite to suppress the intestinal inflammation response to help evade host immunity, making it an ideal candidate to be developed as a novel anti-inflammatory bio-therapeutic. Here we utilized the expression system of the edible green algae *Chlamydomonas reinhardtii* in order to recombinantly produce active TGM1 in a form that could be ingested. *C. reinhardtii* robustly expressed TGM1, and the resultant recombinant protein is biologically active as measured by regulatory T cell induction. When delivered orally to mice, the algal expressed TGM1 is able to ameliorate weight loss, lymphadenopathy, and disease symptoms in a mouse model of DSS-induced colitis, demonstrating the potential of this biologic as a novel treatment of IBD.

## Highlights

• A novel cytokine from a parasitic worm expressed in green algae induces an anti-inflammatory response when fed to mice.
• Recovery from inflammatory colitis is accelerated by the algal-expressed protein.
• Oral administration offers an attractive route for the novel therapy.

## 1 Introduction

Inflammatory Bowel Disease (IBD) is a chronic immunological disorder of the gastrointestinal tract that affects millions of people in the world (de Souza and Fiocchi, 2016, Kaser et al., 2010). Every year, hundreds of thousands of new patients suffer from this disease, with a high risk of becoming seriously sick and debilitated, creating an enormous socioeconomic burden (Burisch et al., 2019). The two principal types of IBD are Crohn's disease (CD) (Baumgart and Sandborn, 2012) and ulcerative colitis (UC) (Danese and Fiocchi, 2011). While diagnosis is well-developed, no curative treatments for these diseases are available, with surgical resection a last resort. IBD can be triggered by poorly understood physiological states, and by numerous environmental factors including diet, stress, and infections. Although the detailed mechanism of IBD development is unclear, it is well acknowledged that IBD is associated with a failure of immune regulatory mechanisms, resulting in chronic inflammation and production of pro-inflammatory cytokines, leading to tissue destruction and tumorigenesis (Burisch et al., 2019, Neurath et al., 2002).

Key inflammatory cytokines have been targeted for IBD treatment, with the most effective therapy found to be antibody against tumor necrosis factor (TNF); however, this is not effective for patients with few immune cells expressing membrane-bound TNF (Wong et al., 2008). An alternative strategy is to deliver recombinant anti-inflammatory cytokines. These include IL-10 (Saraiva et al., 2020), IL-22 (Sugimoto et al., 2008), IL-27 (McLean et al., 2017) and TGF-β (Ihara et al., 2017), which are produced by mucosal immune cells and/or epithelium, which exhibit both potent anti-inflammatory and tissue-repair effects, through regulatory T cell generation and enhancing epithelial cell function. However, the paracrine manner in which these cytokines act, creates a challenge in delivering pharmacologic amounts of these factors only to the gastrointestinal mucosa. In animal models, pathology can be suppressed by pre-treatment with anti-inflammatory cytokines such as IL-10 (Huber et al., 2011), while established disease is ameliorated by oral delivery of IL-10-expressing transgenic *Lactococcus lactis* (Steidler et al., 2000). In clinical trials, systemic IL-10 administration showed no benefit at doses low enough to avoid significant side effects (Buruiana et al., 2010, Schreiber et al., 2000), and published reports on oral delivery have been limited to safety trials in healthy individuals (Braat et al., 2006).

TGF-β has wide-ranging effects in immune suppression and wound repair (David and Massague, 2018) and plays a prominent role in maintaining homeostasis of the mucosal immune system (Ihara et al., 2017, Konkel and Chen, 2011). In colitis, elevated TGF-β expression dampens inflammation, and one therapeutic avenue has been to boost TGF-β efficacy by anti-sense RNA abrogation of the Smad7 inhibitor of the TGF-β signaling pathway (Monteleone et al., 2015). Transgenic *Bacteroides ovatus* which can be induced to release TGF-β have also been shown to downmodulate colitis in mouse models (Hamady et al., 2011).

An alternative strategy to ameliorate inflammatory bowel diseases has emerged from studies with parasitic helminth worms (Weinstock and Elliott, 2013). Globally, there is an inverse relationship between the prevalence of intestinal helminth parasites and the incidence of IBD (Varyani et al., 2017); in mouse models, infections with diverse helminth species can abrogate colitis (Hunter et al., 2005, Leung et al., 2012, Smith et al., 2007), and deliberate infection of IBD patients has been advocated as a new therapy for disease (Summers et al., 2005). One species associated with anti-inflammatory effects *in vivo*, *Heligmosomoides polygyrus*, has been shown to secrete a potent TGF-β mimic, TGM1 (Johnston et al., 2017, Smyth et al., 2018). Parasite TGM1 and mammalian TGF-β have no sequence similarity, however TGM1 ligates the mammalian TGF-β receptor (TBRII), initiating downstream Smad signaling and inducing suppressive Foxp3^+^ regulatory T cell (Tregs) (Cook et al., 2021, White et al., 2021). We are therefore investigating whether oral therapy with helminth-derived TGM, rather than live infection with helminth parasites, would be the therapy of choice for IBD.

In this study, we tested the idea of producing recombinant TGM1 in the edible green algae *Chlamydomonas reinhardtii*, in order to be able to feed TGM1 directly to mice and assess the protein’s action from within the gastrointestinal tract, and with the view to developing a bio-therapeutic for use in humans to treat a range of gastrointestinal inflammatory diseases. Compared to other cell-based expression systems, the use of algae as a cellular factory has several benefits, including cost effective cultivation under photosynthetic growth, and rapid low cost scaling from lab to commercial scale, rendering it orders of magnitude less costly than conventional mammalian cell culture technologies (Dove, 2002). Additionally, unlike other low-cost cell systems such as yeast or bacteria, algae have the cellular machinery in both the endoplasmic reticulum (ER) and chloroplast, that are required for assembling complex mammalian proteins and antibodies (Gregory et al., 2013, Patra et al., 2015, Ravi et al., 2018, Tran et al., 2013). We have previously demonstrated that *C. reinhardtii* can be engineered to express a number of bioactive proteins (Gimpel et al., 2015, Rasala et al., 2012, Tran et al., 2009) and that the biomass can be safely consumed orally in mice and humans (Fields et al., 2020), and shown to be able to deliver recombinant protein cargoes to the intestinal tract of mice (Barrera et al., 2015). We have now expressed an active novel anti-inflammatory cytokine TGM1 in *C. reinhardtii* and further show that the algal TGM1 when given orally is able to regulate immune cells and protect mice from DSS colitis weight loss.

## 2 Materials and methods

### 2.1 Plasmid construction for recombinant TGM1 protein expression

**Fig. 1:**
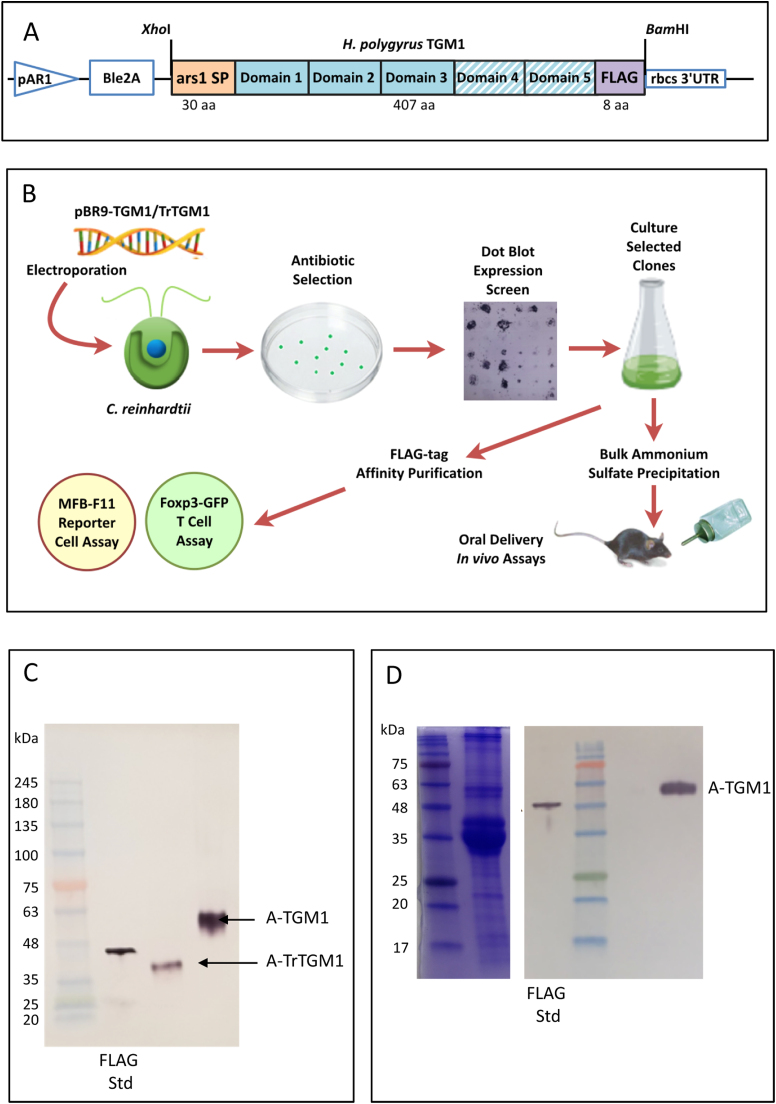
Expression of *Heligmosomoides polygyrus* TGM1 in *Chlamydomonas reinhardtii***.** A. Construct design comprising pAR1, a constitutive promoter (670 bp) used for high level protein expression in *Chlamydomonas*; Ble2A, an antibiotic resistance gene; Ars1 SP, a 30-aa secretion signal peptide (orange filled box) codon-optimized mature 5-domain TGM1 (blue boxes), or truncated TGM1 (TrTGM1, lacking Domains 4 and 5, hatched blue); FLAG tag (purple); and rbcs 3’ UTR (246 bp), a robust element to maintain algal mRNA stability. The sequence encoding the expressed protein was inserted into pBR9 through *Xho*I and *Bam*HI restriction sites as indicated, and is given in full in Supplementary Fig. S1. B. Schematic of experimental approach including dot blot screening to select high expressing clones, and purification of TGM1 by affinity chromatography (C) for *in vitro* bioassays (see Fig. 2) or ammonium sulfate precipitation for *in vivo* administration (see Fig. 3 and 4). C. Western blot of anti-FLAG antibody affinity-purified TGM1 and TrTGM1, stained with Monoclonal ANTI-FLAG® M2-Alkaline Phosphatase antibody. Ten µL of each purified protein sample(TGM1 and TrTGM1) and 50 ng 45-kDa Recombinant Posi-Tag Epitope Tag Protein containing the epitope FLAG tag were loaded onto a SDS-PAGE gel. Marker protein molecular weights are indicated. D**.** Coomassie Blue stained (left hand panel) and anti-FLAG Western blot (right hand panel) of ammonium sulfate-precipitated TGM1. Two µL of TGM1 sample and 150 ng Recombinant Posi-Tag Epitope Tag Protein were loaded onto a SDS-PAGE gel. Marker protein molecular weights are indicated. (For interpretation of the references to color in this figure legend, the reader is referred to the web version of this article.)

To express recombinant TGM1 using *C. reinhardtii*, we designed a codon-optimized TGM1 construct with a FLAG tag based on the previously described secretion vector pBR9 (Rasala et al., 2013), to maximize protein expression (Fig. 1 A). The N-terminal ars1 algae signal peptide allows for the secretion of TGM1 into the medium. Additionally, the AR1 promoter and rbcs 3’ UTR ensure that the transformed construct locates to the nucleus and that protein is continuously expressed under light conditions. pBR9 also encodes the Bleomycin selection gene Ble2A to isolate transformed algae. As previous work had shown that domains 1–3 of TGM1 were sufficient for biological activity (Smyth et al., 2018), a similar construct was made to also express truncated TGM1 (TrTGM1).

The sequence for mature TGM1 (or TrTGM1) was adapted to the nuclear codon usage of *C. reinhardtii* according to the Kazusa DNA Research Institute's database (http://www.kazusa.or.jp/codon/cgi-bin/showcodon.cgi?species=3055) as previously described (Rasala et al., 2013), and is presented in Supplementary Fig. 1. Synthetic genes plus an 8-aa FLAG tag (DYKDDDDK) were synthesized by Genewiz and cloned into pBR9 as *Xho*I/*Bam*HI fragments. The resultant constructs, named pBR9-TGM1 and pBR9-TrTGM1, were transformed into wild type CC-1690 *C. reinhardtii* by electroporation with high-level expressing transformants being selected by dot-blot screening for further large-scale expression and purification (Fig. 1 B).

### 2.2 *C. reinhardtii* transformation and screening

#### 2.2.1 Transformation

*C. reinhardtii* wild type strain CC-1690 was used for secreted TGM1 protein expression. *C. reinhardtii* was grown in tris-acetate-phosphate (TAP) (Gorman and Levine, 1965) liquid medium at room temperature (RT) on a rotary shaker set at 100 rpm, under constant light intensity (100 µmol photons m^−2^ s^−1^) to a concentration of approximately 1 × 10^6^ cells·mL^-1^. Cells were harvested by centrifugation (2000 *g*) and then resuspended with MAX Efficiency® Transformation Reagent for Algae (Thermo Fisher Scientific Inc., MA, Cat #A24229). One million cells in 300 µL transformation reagent were mixed with 5 µg of linearized plasmid digested with *Kpn*I and incubated on ice for 30 min. The cell suspension was placed into a prechilled disposable electroporation cuvette with a 4-mm gap (Bio-Rad Labs., Hercules, CA, Cat #1652081) for 5 min on ice. A BioRad Gene Pulser Xcell with the following settings (Voltage 500 V, Capacity 50 µF and Resistance 800 Ω) was used for electroporation. Electroporated *C. reinhardtii* cells were recovered with 10 mL of 20 mM sucrose in TAP medium for 24 h, and then plated on TAP agar plates with 15 µg·mL^-1^ Zeocin and incubated at RT for 10 days.

#### 2.2.2 Dot blot screening

A dot blot assay (Rupprecht et al., 2010) was modified to screen transformed colonies in order to select clones with higher TGM1 secretion yields. Nitrocellulose membranes, 7 × 7 cm, were marked with a grid of 100 small squares and placed on a 10 cm diameter Fisherbrand™ Petri Dishes (Fisher Scientific, Cat #FB0875713) of TAP agar. One hundred of the transformed of the colonies were randomly picked and patched onto two replicate nitrocellulose membranes in a grid formation and the incubated under light at RT for three days. After three days, all colonies were washed off one of the replicate nitrocellulose membranes with TBST (Tris-buffered saline plus 0.05% Tween-20). The nitrocellulose membrane was blocked with 5% non-fat milk powder in TBST for 1 h and then incubated with Monoclonal ANTI-FLAG® M2-Alkaline Phosphatase antibody (Sigma-Aldrich, St. Louis, MO, USA, Cat #A9469; final dilution 1:5000) for 1 h. The membrane was then washed with TBST 3 × 5 min and visualized in nitro-blue tetrazolium (NBT) / 5-bromo-4-chloro-3’-indolyphosphate p-toluidine salt (BCIP) (Sigma-Aldrich, St. Louis, MO, USA, Cat #B5655) added directly to the membrane for development. Developed dot blot intensity was correlated with yield of secreted TGM1 and the transgenic clones with highest protein secretion were recovered from the second replicate membrane for further protein expression and purification.

#### 2.2.3 Western blot and Coomassie Blue staining

Transgenic TGM1 strains were grown in liquid medium TAP. Cells were cultured at RT, on rotary shaker set at 100 rpm, under constant light intensity (100 µmol photons m^−2^ s^−1^), to a cell concentration around 6 × 10^6^ cells mL^-1^. Supernatants were harvested by centrifuging cells at 7800 *g*, added to 4 volumes of prechilled acetone (Sigma-Aldrich Cat # 650501) and incubated at 4 °C overnight. Precipitated protein pellets were obtained by centrifuging acetone-treated supernatants at 17,000 *g* for 10 min at 4 °C and air dried for 5 min. ddH_2_O was used to resuspend the dried protein pellet and 4X Laemmli sample buffer (BioRad, Cat # 1610747) with 10% β-mercaptoethanol (Sigma-Aldrich Cat # M6250) was then added as 1:4 ratio (sample buffer: sample). Reduced samples were heated at 65 °C for 10 min and loaded onto 10% Mini-PROTEAN® TGX™ Precast Protein Gels (BioRad, Cat # 4561036). The molecular weight marker used was AccuRuler RGB Plus Prestained Protein Marker (Biopioneering, Cat # PM-001). A voltage of 120 was applied and the gels were run for 1 h. One gel was stained in Coomassie SimplyBlue™ SafeStain (Thermos Fisher Scientific, Cat # LC6060) for 1 h, followed by destaining in ddH_2_O. Another gel was transferred to nitrocellulose membrane (BioRad, Cat # 1620112). The membrane was blocked with 5% non-fat milk in TBST for 1 h at RT. The membrane was probed with Monoclonal ANTI-FLAG® M2-Alkaline Phosphatase antibody as described above for 1 h and then washed with TBST 3 × 5 min; NBT/BCIP developer tablet (Sigma FAST B5655, St. Louis, MO, USA) was dissolved in 50 mL ddH2O and used to visualize the protein band on the membrane.

### 2.3 *C. reinhardtii* bulk expression conditions for algal TGM1

Transgenic TGM1 and TrTGM1 strains were grown with 1 L TAP liquid medium in 2 L Erlenmeyer flasks. These culture flasks were maintained at RT on a rotary shaker set at 100 rpm, under constant light intensity (100 µmol photons m^−2^ s^−1^), to a cell concentration of approximately 6 × 10^6^ cells mL^−1^. Supernatants were harvested by centrifugation (7800 *g*) and then filtered by Whatman Grade GF/A Fine Retention Filter paper (GE Healthcare Life Science, 1820-021). Filtered supernatant was further processed to obtain recombinant protein with either FLAG affinity purification or ammonium sulfate precipitation (Fig. 1 C, D). Wild type algae strain CC-1690 was grown under the same conditions and treated in the same manner as the transgenic TGM1 strain and used as an algal protein control for assays and *in vivo* mouse models.

### 2.4 Purification of secreted algal TGM1 from *C. reinhardtii*

#### 2.4.1 Anti-FLAG resin purification of algal TGM1 and TrTGM1

FLAG affinity chromatography was used to purify TGM1 and TrTGM1 for mass spectrometry, glycosylation analysis and *in vitro* cell-based assays for activity. Briefly, 1 mL of anti-FLAG affinity gel (Sigma-Aldrich Cat # A4596) was equilibrated in lysis buffer (50 mM Tris, 150 mM NaCl, 1 mM EDTA, pH 7.4) with 5 column volumes (CV) and then loaded with 2 L filtered supernatant. The resin was washed with 5 CV of lysis buffer and then eluted with 6 sequential 1 CV of elution buffer (100 mM Glycine, 400 mM NaCl, pH 3.5). The pH of the eluate was immediately adjusted to pH 7.5 with 50 µL 1 M Tris-HCL. For quantification, 10 µL of purified TGM1 and TrTGM1, and 50 ng 45-kDa recombinant Posi-Tag Epitope Tag Protein containing the epitope FLAG tag(BioLegend, Cat #931301) were loaded to SDS-PAGE gel and analyzed by Western blot with anti-FLAG antibody; band intensities were then quantified by Image Studio Lite(LI-COR Biosciences, Lincoln, NE, USA). The FLAG-purified TGM1 was then sent for mass spectrometry, glycosylation analysis and both FLAG-purified TGM1 and TrTGM1 were used for *in vitro* cell-based activity assays.

#### 2.4.2 Ammonium sulfate precipitated TGM1 and wild type algal proteins for *in vivo* mouse models

For larger scale isolation of TGM1, ammonium sulfate was added slowly to filtered algae supernatant to a final salt concentration of 250 g·L^-1^. The mixture was incubated at 4 °C overnight and then centrifuged at 7800 *g* to harvest the precipitated protein pellet. The precipitated protein pellet was resuspended in DPBS (Thermo Fisher, Cat #14190144) and dialyzed twice with Thermo Scientific™ SnakeSkin™ Dialysis Tubing (10 K MWCO, Thermo Fisher, Cat # 68100) overnight in DPBS at 4 °C. To protect proteins from aggregation and increase their stability sterile glycerol was added into the samples with a final concentration of 2% (v/v). For quantification, 2 µL of resuspended protein supernatant and 150 ng of recombinant Posi-Tag Epitope Tag Protein as a FLAG standard were analyzed by SDS-PAGE gel and protein band pixel intensities were quantified by Image Studio Lite. Total protein concentrations in precipitated supernatant were quantified by Pierce™ BCA Protein Assay Kit (Thermo Fisher, Cat #23225) at 562 nm against BSA standards.

Wild-type (WT) algal proteins used as a control in assays and mouse models was CC-1690 *C. reinhardtii* WT filtered supernatant processed with the same procedure as above and the final concentration of these supernatants were measured by Pierce™ BCA Protein Assay Kit.

### 2.5 Glycosylation analysis and mass spectrometry of FLAG-purified algal TGM1

For glycosylation analysis, 1 µg FLAG-affinity purified algal TGM1 samples were digested with either 5 U of PNGase F (NEB, Cat # P0704S) or 5 U EndoH (NEB, Cat # P0702S) at 37 °C for one hour. Digested proteins were heat inactivated at 65 °C for 10 min and analyzed by SDS-PAGE. For more detailed investigation, untreated protein samples in 50 mM ammonium bicarbonate, 0.1% w/v ProteaseMax surfactant were reduced and alkylated prior to proteolytic digestion with trypsin at a ratio of 1:50. Proteolysis was stopped, and surfactant hydrolyzed by the addition of 0.5% trifluoroacetic acid (TFA). Sample desalting was performed using HyperSep Hypercarb solid‐phase extraction tips followed by drying by vacuum centrifugation (Eppendorf).

Liquid Chromatography with tandem mass spectrometry (LC MS/MS) was performed by nano-flow liquid chromatography (U3000 RSLCnano) coupled to a hybrid quadrupole-orbitrap mass spectrometer (Q Exactive HF). Peptides were separated on an Easy-Spray C18 column (75 µm × 50 cm) using a 2-step gradient from 97% solvent A (0.1% formic acid in water) to 10% solvent B (0.08% formic acid in 80% acetonitrile) over 5 min then 10–50% B over 75 min at 300 nL.min^-1^. Mass spectra were acquired with automated data‐dependent switching between full‐MS and tandem MS/MS scans using stepped collision energy. Data analysis was performed using BioPharma Finder 4.0. All materials and equipment were supplied by Thermo Fisher Scientific unless otherwise stated.

### 2.6 *In vitro* cell based assay for recombinant TGM1 bioactivity

#### 2.6.1 TGF-β bioassay

The TGF-β bioassay (MFB-F11) developed by Tesseur et al. (2006) was performed as previously described (Smyth et al., 2018). Briefly, confluent MFB-F11 cells were added to each well of a 96-well round-bottomed plate. Wild type algal proteins or log dilutions (starting at 100 ng·mL^-1^ of purified proteins such as HEK293T-expressed TGM1 (expressed and purified as previously described (Johnston et al., 2017)) or algal-expressed TGM1/TrTGM1 were then added to each well in a volume of up to 50 µL and incubated for 24 h at 37 °C in an atmosphere of 5% CO_2_. Subsequently, 20 µL of supernatant was aspirated from each well, added to an ELISA plate (NUNC) with 180 µL of reconstituted Sigma FastTM *p*-nitrophenyl phosphate substrate and incubated at RT in the dark for up to 4 h. Plates were read on at 405 nm on an Emax precision microplate reader (Molecular Devices). All conditions were set up in duplicate and repeated at least twice.

#### 2.6.2 Foxp3^+^ Treg induction assay

As described previously (White et al., 2021), a single cell suspension was prepared from the spleens of naïve Foxp3-GFP BALB/c transgenic mice (Fontenot et al., 2005), with contaminating red blood cells removed by resuspending the cells from one spleen in 2 mL of red blood cell lysis buffer (Sigma) and incubating at RT for 2 min. Cells were then washed and resuspended in RPMI medium supplemented with 2 mM L-glutamine (Gibco), 100 U·mL^-1^ penicillin (Gibco), 100 μg·mL^-1^ streptomycin (Gibco), 10% heat-inactivated fetal calf serum (FCS) (Gibco), and 1x MEM non-essential amino acid solution (Gibco). CD4^+^ T cells were enriched by magnetic sorting using the mouse naive CD4^+^ T cell isolation kit (Miltenyi 130-104-453) on an autoMACS Pro Separator (Miltenyi) as per manufacturer’s instructions. Cells were cultured at 4 × 10^5^ per well in flat-bottomed 96-well plates (Costar) pre-coated with 10 µg·mL^-1^ of anti-CD3 (clone 145-2C11; Invitrogen) with the addition of IL-2 (Miltenyi) at a final concentration of 400 U·mL^-1^ and with or without retinoic acid (Sigma) at a final concentration of 1 nM. Purified mammalian TGF-β (Peprotech), HEK293T expressed TGM1 (expressed and purified as previously described, (Johnston et al., 2017)) or algal TGM1/TrTGM1 proteins were added at a concentration of 20–100 ng·mL^-1^ and cells cultured at 37 °C in 5% CO_2_ for at least 72 h before being removed for flow cytometric analysis. All conditions were set up in triplicate and repeated at least twice.

### 2.7 Flow cytometric analysis

For viability staining, LIVE/DEAD® fixable blue (Life Technologies) was diluted to 1:1000 in PBS; 100 µL was added to each sample of cells, which were then incubated in the dark for 20 min at 4 °C and washed twice in FACS buffer. To prevent non-specific antigen binding, cells were incubated with 50 µL of polyclonal IgG (Sigma) (diluted 1:50 in FACS buffer) for 10 min at 4 °C and then washed twice in FACS buffer. Cells were surface stained with anti-CD3-BV711 (17A2, Biolegend), anti-CD4-PerCP/Cy5.5 (GK1.5, BioLegend) and anti-CD25-BV650 (PC61, Biolegend) to a total volume of 50 µL (diluted 1:200). Separate Foxp3 staining was not always required as cells were from Foxp3-GFP transgenic mice however; to assess transcription factor expression, cells were stained with anti-Foxp3-eF450 (FJK-16s, eBioscience) and anti-RORγt-PE (Q31–378, BD Biosciences), both at 1:100 dilution, using the Transcription Factor Staining Buffer set (eBioscience) and following manufacturer’s instructions. Single stain controls were individually added to one drop of UltraComp eBeads (eBioscience). Samples were incubated for 20 min at 4 °C, washed twice in FACS buffer and then resuspended in 200 µL FACS buffer. All samples were acquired on a BD Biosciences Celesta and analyzed using FlowJo software (Tree Star).

### 2.8 Animal models

Female 6–8 week old C57BL/6J mice were purchased from Envigo (Huntingdon, UK) and housed in specific pathogen-free conditions and acclimatized in the animal unit for at least 1 week after arrival before experimental models were set up. All animal work was approved by the Ethical Review Board of the University of Glasgow and procedures were performed under a UΚ Home Office licence.

#### 2.8.1 Mouse feeding trial

For initial evaluation of algal expressed TGM1 on mouse health and whether it would be tolerated/remain active after being ingested, we added algal expressed recombinant TGM1 protein directly to drinking water and allowed mice to drink ad libitum. Body weight and food/water intake of the mice were monitored daily. For the control group, WT *C. reinhardtii* algal proteins or PBS/ 2% (v/v) glycerol were added to drinking water at the same volume and timing as the algal TGM1. Mice were sacrificed and isolation of lymphocytes from the Peyer’s patches (Mosconi et al., 2015), mesenteric lymph nodes (MLNs) (White et al., 2021) lamina propria of small (Webster et al., 2020) and large (Clay et al., 2020) intestines were analyzed by flow cytometry.

#### 2.8.2 DSS colitis model

The dextran sodium sulfate (DSS) chemical model of mouse model of colitis (Chassaing et al., 2014) was selected to determine the effects of algal expressed TGM1 in a murine model of acute colitis. Dextran sodium sulfate (36,000–50,000 MW, MP Biomedicals, Santa Ana, California, USA) was added to the drinking water at 2% (w/v) for a total of 4 days to disrupt the gut mucosal barrier, resulting in acute colitis. Mice were then given normal drinking water for the remainder of the experiment in order to monitor weight recovery. *C. reinhardtii* expressed TGM1 (ammonium sulfate precipitated from the supernatant) was also added to the drinking water initially throughout the 4-day DSS administration and for an additional 3 days (equaling a total of 7 days of algal TGM1 administered to the mice).

Mice were weighed and scored daily for the entirety of the experiment using a Disease Activity Index (DAI) matrix which comprised of scores out of 4 for % weight loss, blood, stool consistency and general appearance. Each day scores for each parameter were summed to give a DAI total out of 16 for each mouse. Animals were euthanized if they lost greater than 20% of their initial weight. Peyer’s patches and mesenteric lymph nodes were isolated for analysis by flow cytometry.

### 2.9 Statistical tests

Graphs and statistics were analysed using Prism (GraphPad, San Diego, California, USA). Shown are the means ± standard error (SEM), and one-way or two-way ANOVA or *t-*tests (paired or unpaired) were used where appropriate, with non-parametric Kruskal-Wallis tests being applied if data were not normally distributed. *P < 0.05.

## 3 Results

### 3.1 Expression and glycan analysis of *C. reinhardtii* expressed TGM1

To express recombinant forms of the parasite TGF-β mimic TGM1 and TrTGM1 using *C. reinhardtii*, we chose to utilize a vector (pBR9) that would insert the gene cassette into the nucleus and secrete the recombinant protein continuously under light conditions (Fig. 1 A). Our strategy of expression included screening recombinant algae for high-expressing clones by a modified dot-blot assay, and purification of TGM1 proteins *via* a FLAG-tag suitable for antibody affinity chromatography (Fig. 1 B). This allowed us to test algal expressed TGM1 functionally, comparing it to the mammalian (HEK293T) expressed TGM1.

For both constructs, single bands were detected in the algal supernatant by anti-FLAG Western blot (Fig. 1 C). Quantification of the recombinant TGM1 against FLAG standard showed that the recombinant protein accumulated to approximately 5% of total soluble protein in the supernatant (Fig. 1 D, SupplementaryTable S1). Algal TGM1 was affinity purified using anti-FLAG M2 affinity resin, and the TGM1 amino acid sequence was confirmed using mass spectrometry (Supplementary Fig. 2A).

We next assessed the level of glycosylation of the recombinant TGM1 expressed by C. *reinhardtii*, as potential modification with plant-specific glycans, including core β1,2-xylose and α1,3-fucose, are reported to cause immunogenicity of recombinant proteins and evoke human antibody responses (Bardor et al., 2003). We subjected FLAG-purified algal TGM1 to digestion with PNGase F and Endoglycosidase H. Only PNGase F appeared to show minor changes to the FLAG-purified algal TGM1 shown by the slight laddering of the protein on SDS-PAGE analysis after digestion (Supplementary Fig. 2B). Algal TGM1 was subjected to further glycosylation analysis by mass spectrometry, identifying 4 sites of *N*-glycosylation, and 1 of *O*-glycosylation (Supplementary Table S1); one potential *N*-glycosylation site (N-318 of the mature protein, Supplementary Fig. 2) was not utilized. Although two previous studies found that 10–20% of glycosylated cellular proteins from *C. reinhardtii* carried one or two xylose additions to the mature oligomannoside chain (Lucas et al., 2020, Mathieu-Rivet et al., 2013), we could not detect any xylosylation of the recombinant TGM1 protein. In addition, we did not detect plant specific core α1,3-fucose on recombinant TGM1, consistent with this modification occurring in a core xylose-dependent manner (Oltmanns et al., 2019). Overall, only mammalian-like *N-*glycans were detected by our mass spectrometry analysis, and the PNGase F digestion (Supplementary Fig. 2B) indicated that their abundance was at a very low level.

### 3.2 Bioactivity of TGM1 expressed in recombinant algae

**Fig. 2:**
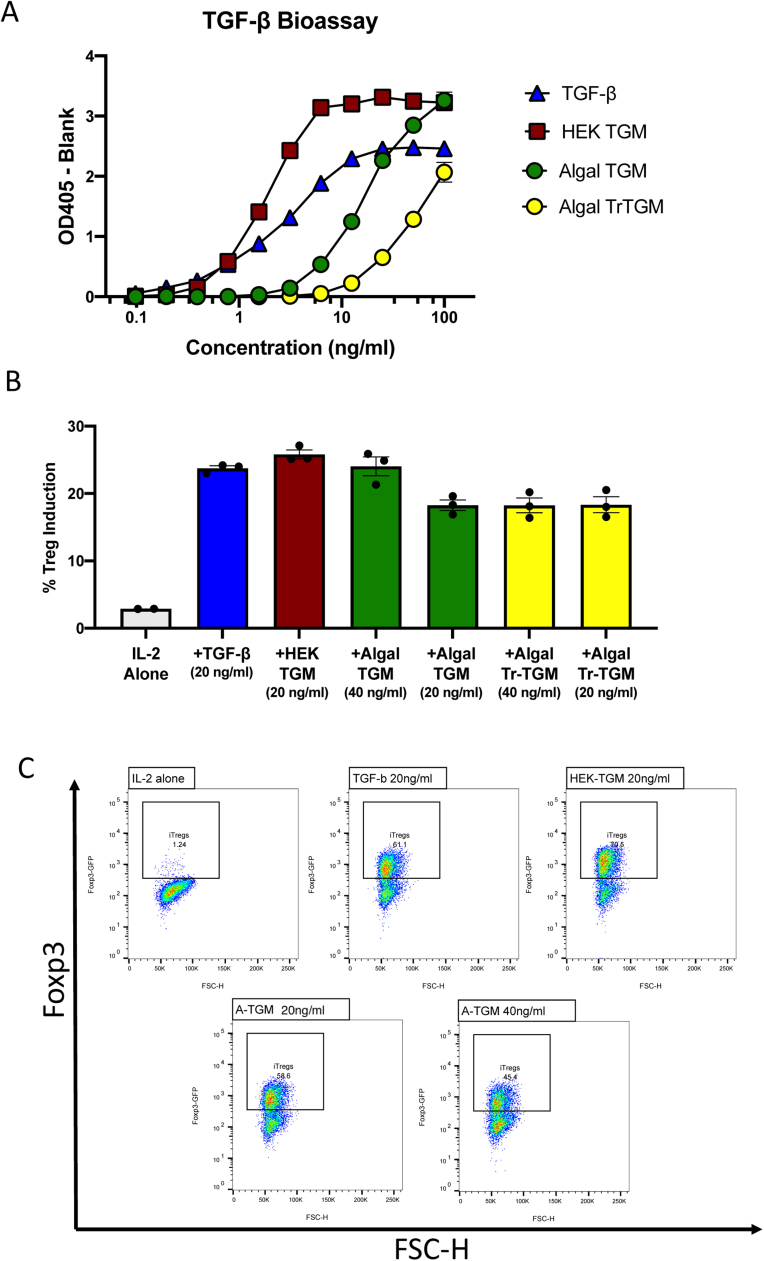
FLAG-purified algal TGM1 and its bioactivity on TGF-β-responsive cell assays. A. MFB-F11 TGF-β receptor binding assay. MFB-F11 cells encode a Smad-responsive reporter construct which induces secretion of alkaline phosphatase following ligation of the TGF-β receptors; alkaline phosphatase is detected by addition of *p*-nitrophenyl phosphate substrate generating a color reaction. Cells were incubated with mammalian TGF-β, HEK293 expressed TGM1, οr algal secreted TGM1 / ΤrTGM1 at the indicated concentrations and separately for 24 h, before recovery of supernatants for enzyme assay with *p*-nitrophenyl phosphate substrate. Data represent one of two similar experiments. B. Naïve T cells from transgenic mice encoding a Foxp3-GFP fusion protein (Fontenot et al., 2005) respond to TGF-β receptor ligation by expression of GFP that is detected by flow cytometry. Induction of primary mouse splenic CD4^+^ T cells to Foxp3-GFP expression. Data represent one of two similar experiments. C**.** Representative flow cytometry plots of Foxp3-GFP reporter T cells treated with TGF-β or TGM1 from mammalian (HEK) or algal (A-TGM) expression systems, together with IL-2. Control cultures received IL-2 alone. Forward Scatter (FSC-H) and Foxp3 expression and are plotted on the X and Y axes, with the same gate for positive cells for all samples.

We next tested FLAG-purified recombinant TGM1 and TrTGM1 produced in *C. reinhardtii* in two *in vitro* assays, a TGF-β reporter bioassay using MFB-F11 cells in which Smad signaling drives plasmid-encoded secretory alkaline phosphatase (Tesseur et al., 2006), and a Treg induction assay in which naive Foxp3-negative mouse splenocytes from transgenic mice encoding a Foxp3-GFP fusion protein, are induced to express Foxp3-GFP (White et al., 2021). In the reporter assay, the algal-expressed cytokines showed bioactivity (Fig. 2 A), while wild-type algal products showed no effects (Supplementary Fig. 3 A). We noted, however, that the full-length algal TGM1 was approximately ten-fold less potent than the equivalent expressed in mammalian HEK293T cells. Nevertheless, at higher concentrations, algal TGM induced a stronger signal than even mammalian TGF-β. In addition, we found that the truncated TGM-1, lacking domains 4 and 5, was significantly weaker than the full-length expressed in the same algal system when tested in the MFB-F11 system, although at the highest concentrations it was able to equal the signal induced by the mammalian cytokine.

We next evaluated whether algal-produced TGM1 proteins could induce Foxp3^+^ Treg cells from naïve pre-sorted Foxp3-negative CD4^+^ T cells. Cell cultures were treated with mammalian TGF-β**,** HEK293T-expressed TGM1, or FLAG-affinity-resin purified algae TGM1, together with the growth factor IL-2. Three days later, flow cytometry was used to identify induced Foxp3-GFP^+^ Treg cells (Fig. 2 B). In this system, both algal recombinant TGM1 and TrTGM1 proteins were able to induce Foxp3 to similar percentages as compared to TGF-β, and mammalian expressed TGM1, with the induction level being well above the background signal for the control group incubated with IL-2 alone (Fig. 2 C). Because retinoic acid (RA) is a known enhancer of Foxp3 induction, similar assays were conducted with and without RA, but we found no significant increase in its presence with the different TGM preparations (Supplementary Fig. 3B).

### 3.3 Increase in mucosal Treg cell numbers following oral delivery of TGM1 to mice

We then explored whether purified algal TGM1 could be fed and tolerated by mice and if so, could induce any immunological changes in intestinal tissues. In view of the lower potency and reduced quantities available of the truncated TGM1 protein, these studies focused on the full-length construct. We opted to administer TGM1 by addition of the whole algal secreted products to drinking water (at 100 µg·mL^-1^ TGM1 within a total of 5.2 mg·mL^-1^, Supplementary Table S1) to provide carrier protein, and allow comparison with wild-type algal secretions. We then monitored water and food intake, and body weights, over time (Supplementary Fig. 4).

Water intake was first ascertained and found to increase marginally above the average mouse consumption of 5 mL per day (indicated on Supplementary Fig. 4 A); this equates to mice receiving approximately 0.5 mg of the algal TGM1 per day. Control mice received water with an equivalent concentration of WT algal secreting proteins including a matching volume of PBS/2% glycerol used in adding TGM1 to drinking water. Over the course of an 8-day period, there was no significant difference measured between the control group with WT algae and group receiving the algal TGM1 water, with respect to water or food intake (Supplementary Fig. 4A, B) or weight (Supplementary Fig. 4C) indicating that algal TGM1 consumed in drinking water showed no detrimental effects on appetite or body weight.

**Fig. 3:**
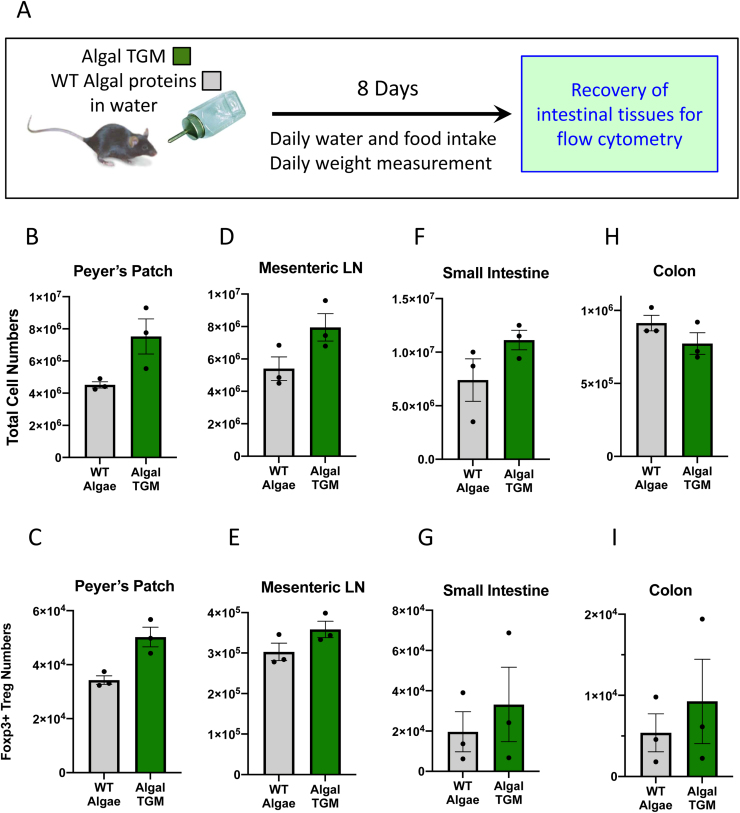
Effect of algal TGM1 on mucosal immune cell populations. A. Experimental design for algal TGM1 feeding trial. B–I. Cell counts and Foxp3^+^ Treg numbers in intestinal tissues following feeding of WT algal proteins or algal-expressed TGM1, in the Peyer’s Patches (B, C), Mesenteric Lymph Nodes (D, E), Small Intestine (F, G) and Colon (H, I). Bars represent means and standard errors, with data from individual mice shown as solid symbols. Data represent one of two similar experiments.

At day 8, mucosal tissues (Peyer’s patches, mesenteric lymph nodes, small intestine, colon,) were harvested and cells were isolated, enumerated and subjected to flow cytometric analysis (Fig. 3 A). Peyer’s patches are the specialized structures in the small intestinal epithelium that act as the first-line lymphoid organ for mucosal immune system, in which lymphocytes activated by epithelial dome microfold (M) cells that transport antigens from the intestinal lumen (Da Silva et al., 2017). We noted with interest that both total cell counts, and the numbers of Foxp3^+^ Tregs, showed substantial increases in mice receiving TGM (Fig. 3 B, C), although the frequency of Tregs within the total populations showed little change. Broadly similar patterns were observed in the mesenteric lymph nodes (MLNs, Fig. 3 D, E), and in intestinal tissue preparations from the small intestine (Fig. 3 F, G) and large intestine (Fig. 3 H, I), although in the latter case total cell numbers declined slightly, resulting in an overall increase in Treg proportions.

### 3.4 Orally delivered algal TGM1 protects mice from DSS colitis weight loss

**Fig. 4:**
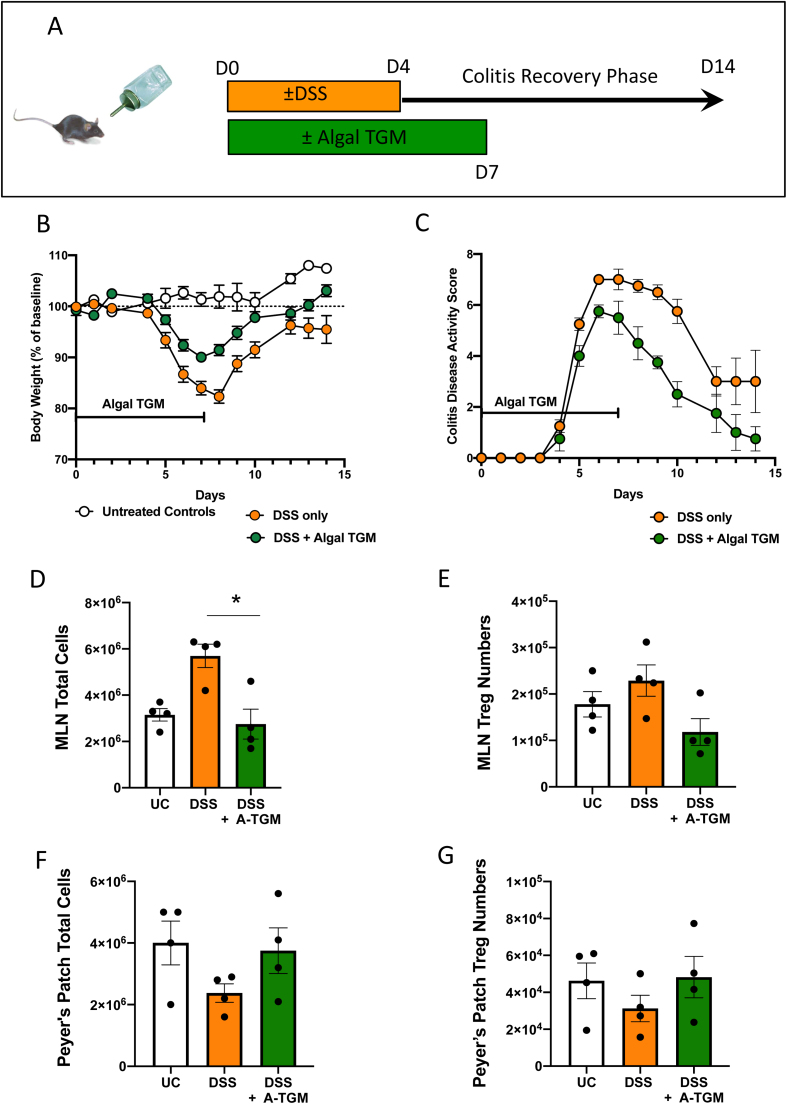
Amelioration of *in vivo* DSS mouse colitis with orally administered algal TGM1. A. Experimental design for DSS mouse feeding trial. DSS was fed to mice in the drinking water for 4 days with additional algal TGM1, followed by 3 days of standard drinking water with algal TGM1. The mice were allowed to recover for a further 7 days with standard drinking water only. B, C. Course of colitis in mice receiving DSS in drinking water, measured by (B) Weight change, and (C) Disease Activity Scores, in mice fed with algal TGM1, and controls receiving DSS alone. D–G. Total cells and Foxp3^+^ Treg numbers in MLNs (D, E) and Peyer’s Patches (F, G) counted by flow cytometry. Bars represent means and standard errors, with data from individual mice shown as solid symbols. Data is from a single experiment.

To investigate anti-inflammatory effects of algal TGM1 in an *in vivo* mouse model of immunopathology, we used dextran sodium sulfate (DSS), a chemical to induce mucosal epithelial damage and disrupt mucosal barrier function (Chassaing et al., 2014). A percentage of 2% DSS (weight/volume) was added to the drinking water, resulting in an acute colitis, and the water for the algal TGM group contained 100 µg·mL^-1^ TGM1 in addition to DSS. Mice were then allowed to drink freely from the DSS water for a 4 day period followed by a recovery period of 3 days on either standard drinking water (control group) or drinking water plus 100 µg·mL^-1^ TGM1 (algal TGM1 group), and an additional 7 days on standard drinking water to allow for mucosal repair (Fig. 4 A). Control mice were given the equivalent volume of wild-type algal proteins or diluent (1 x PBS/ 2% glycerol) in place of algal TGM1. Each mouse was weighed and scored daily to determine if algal TGM1 mitigated the weight loss effect of DSS.

All DSS mice started losing weight after administration of DSS, however mice administered algal TGM1 were protected from dramatic weight loss during DSS treatment and returned to their weight levels as measured before DSS treatment (Fig. 4 B). Compared to mice given DSS alone, the group also receiving wild-type algal proteins showed no protection, and for clarity data from this group are not shown. In the absence of algal TGM1, mice suffered a 20% weight loss 3–4 days after DSS administration while the weight loss of DSS + algal TGM1 group was only 10% in the same time window. In addition, mice not receiving TGM1 could not fully recover their weight loss, and continued to exhibit a 10% deficit after switching back to normal drinking water (Fig. 4 B). In parallel with attenuated weight loss, algal TGM1 also ameliorated disease symptoms accompanying DSS administration, reaching a lower severity and returning closer to baseline more quickly (Fig. 4 C). Taken together, these results indicate that algal TGM1 is effective at protecting mice from DSS-induced intestinal injury and weight loss.

DSS-induced intestinal inflammation is known to provoke lymphadenopathy with significant increases in the size, weight, and cellular content of the mesenteric lymph nodes (MLNs) (Rehal et al., 2018). Consistent with this previous work, we also found that total MLN cell numbers were greatly increased in mice treated with DSS, but remained at normal levels in the algal TGM recipients (Fig. 4 D). MLN Treg numbers were not higher in TGM-treated mice (Fig. 4 E) which may reflect more intense inflammation in the DSS alone animals. Interestingly, DSS treatment reduced Peyer’s patch numbers (Fig. 4 F, G) although not in those mice receiving TGM, suggesting a protective effect. We also investigated stimulation of RORγt+ T cells, which are associated with gut inflammation and increases permeability of the epithelial barrier. There was an increase in all DSS-treated mice seen in the MLN (Supplementary Fig. 5B) but not the Peyer’s patches (Supplementary Fig. 5C).

## 4 Discussion

Immuno-modulatory parasites are increasingly recognized as treasure chests of novel, biologically active products with therapeutic potential, particularly for inflammatory disorders which are increasingly prevalent in industrialized societies (Johnston et al., 2009, Kahl et al., 2018, Maizels et al., 2018). Among the most severe of these “diseases of modernity” are inflammatory bowel diseases, for which no curative therapy is yet available (Kaser et al., 2010). The discovery of the immune suppressive protein, TGM1, which mimics TGF-β, from a helminth parasite which itself can suppress intestinal inflammation (Leung et al., 2012), led us to test the ability of recombinant TGM1 to inhibit inflammatory bowel disease in the well-characterized mouse model of DSS. Because the parasite, *H. polygyrus*, resides in the intestinal lumen, we reasoned that oral delivery would replicate its natural pathway of host modulation; and to test this hypothesis, we produced quantities of the TGM1 protein in the alga *C. reichardtii.*

Expression of recombinant proteins in algae has been reported to be a cost-effective method to produce protein biologics, including human therapeutics such as antibodies, growth hormones, cytokines, and vaccines (Gimpel et al., 2015, Gregory et al., 2013, Patra et al., 2015, Rasala et al., 2012, Ravi et al., 2018, Tran et al., 2009). Algae have also been shown to be an effective vehicle to deliver vaccines (Gregory et al., 2013) and recombinant therapeutic proteins (Barrera et al., 2015) to the gut, as *C. reinhardtii* has been granted GRAS status by the FDA and is safe to consume (Fields et al., 2020). We therefore tested expression of TGM1 in this expression system and found that it produced sufficient quantities of soluble, mature recombinant TGM1 protein. We then used two established assays for TGF-β function which confirmed fully functional properties for the recombinant TGM1, including the induction of the Foxp3^+^ phenotype of immunosuppressive regulatory T cells (Tregs). We noted that algal TGM1 required a higher concentration for equivalent effect to TGM1 produced in mammalian cells, but that is nevertheless reached a greater maximum signal than did TGF-β itself.

To evaluate the safety and physiological effect of algal TGM1 at steady state, it was administered to mice in the absence of any other intervention. No adverse effects were found, and there was a generalized enhancement of immune cell numbers, including Tregs, which was most marked in the Peyer’s patches, the localized immune structures in the small intestinal wall that serve as the primary interface between luminal contents and the mucosal immune system.

We then tested TGM1 in the DSS induced model of colitis, in which mice exhibit immunopathogenic phenotypes including weight loss, diarrhea and rectal bleeding, resembling clinical symptoms of human disease (Okayasu et al., 1990, Wirtz et al., 2007). Along with the destruction of mucosal layer by DSS, commensal bacteria can cross the impaired epithelial barrier, stimulating the intestinal immune system to secrete pro-inflammatory cytokines and triggering inflammation. One corollary of intestinal inflammation is enlargement of the mesenteric lymph nodes which we observed were greatly increased in DSS-treated mice but not in those also receiving algal-expressed TGM orally. This suggests that TGM1 is able to reduce the inflammatory response of immune cells in this model.

Although TGM1 given to mice at steady-state tended to increase Treg numbers, a different outcome was observed in the DSS colitis model. One possibility is that inflammation itself elicits an influx of Tregs, such that at certain times they may be more abundant than in non-diseased cases; in IBD patients for example there are fewer circulating Tregs, but more in inflamed mucosal tissues (Maul et al., 2005, Wang et al., 2011). An alternative explanation is that the disease suppresses the *de novo* (TGF-β-dependent) induction of Tregs, for example through increased inflammatory cytokine production and/or loss of dendritic cell subsets able to drive Treg expansion (Boschetti et al., 2017). It will be important in future studies of TGM1 modulation to tease out its effects on dendritic cells which play critical roles in driving and balancing intestinal inflammation (Hart et al., 2005).

Over recent years, a number of potential new targets for IBD therapies have been explored. For example, DCs and macrophages in IBD patients produce a higher level of activated IL-1; unfortunately however, antibody against IL-1 has no benefit for IBD treatment (Kojouharoff et al., 1997). Similarly, a monoclonal antibody targeting IFN-γ had no effect on Crohn’s disease patients (Reinisch et al., 2006). In the case of UC, patients express a more Type 2 profile (Fuss et al., 1996, Heller et al., 2005, Neurath, 2014), but anti-IL-13 antibody tralokinumab was not reported to have shown great clinical activity in patients with UC (Danese et al., 2015). For patients with either Crohn’s disease or UC, Th17 cells are abundant in inflamed intestine terminal ileum and produce IL17A, IL17F, IL26 and IFNγ. Nevertheless, treatment of secukinumab, an antibody to IL17A, was ineffective for Crohn’s disease and had severe side effects (Hueber et al., 2012). The most effective strategy to target pro-inflammatory cytokine is antibody against TNF which unfortunately is not effective for patients with few immune cells expressing membrane-bound TNF (Wong et al., 2008).

In contrast, treatment with recombinant anti-inflammatory cytokines has not been widely reported in IBD patients. Here we show for the first time that an algal produced TGF- β mimic, TGM1, acts in a similar manner to mammalian expressed TGM1 and limits disease intensity and lymph node inflammation when given orally in mouse model of colitis. In the case of TGF-β, caution has been expressed as the mammalian cytokine is associated with Th17 differentiation when IL-6 is present *in vitro* (Veldhoen et al., 2006). However, recently TGF-β has been observed to be associated with a non-inflammatory Th17 subset (Lee et al., 2020), and our own recent work demonstrates that TGM1 is effective in inducing Foxp3 expression in human Th17 memory cells (Cook et al., 2021) In addition, with respect to concerns that TGFβ promotes tissue fibrosis, TGM1 has been shown to be significantly less fibrogenic than mammalian TGF-β (Johnston et al., 2017).

In conclusion, among a number of potential therapeutic cytokine therapy strategies, recombinant algal TGM1 offers several advantages including the oral mode of delivery, allowing direct action on inflamed mucosal surfaces while minimizing any systemic off-target effects. It is also very economical, compared to the cost of anti-TNF therapy and limited efficiency of other anti-inflammatory cytokines. It may also be beneficial to identify if recombinant algal TGM1 can be combined with other therapeutics, perhaps also expressed in algae, or antibodies against other immunomodulatory cytokines (for example TNF) which may further increase their therapeutic efficacy for patients with diverse immunopathological backgrounds.

## CRediT authorship contribution statement

**Danielle J. Smyth:** Conceptualization, Writing – review & editing. **Bijie Ren:** Writing – review & editing. **Madeleine P.J. White:** Writing – review & editing. **Caitlin McManus:** Writing – review & editing. **Holly Webster:** Writing – review & editing. **Vivien Shek:** Writing – review & editing. **Caroline Evans:** Writing – review & editing. **Jagroop Pandhal:** Supervision, Funding acquisition, Writing – review & editing. **Francis Fields:** Conceptualization, Writing – review & editing. **Rick M. Maizels:** Conceptualization, Supervision, Funding acquisition, Writing – review & editing. **Stephen Mayfield:** Conceptualization, Supervision, Funding acquisition, Writing – review & editing.

## Declaration of Competing Interest

The authors declare the following financial interests/personal relationships which may be considered as potential competing interests: SPM has an equity position in Algenesis Inc a company that uses algae biotechnology to make renewable polymers from algae.
