# Supplementary material for: Oral delivery of a functional algal-expressed TGF-β mimic halts colitis in a murine DSS model
Source: J Biotechnol. 2021 Nov 10;340:1–12. doi: 10.1016/j.jbiotec.2021.08.006 (PMC8516079; doi:10.1016/j.jbiotec.2021.08.006)

Supplementary Material

Supplementary Figure Legends

Supplementary Figure 1.

Nucleotide sequence of TGM1 as codon optimized for *C. reinhardtii* nuclear transcription. The *C. reinhardtii* ars1 Signal Peptide secretion sequence (90bp, 1-90, in orange) immediately precedes the 5-domain TGM1 mature sequence, labeled in blue, with the darker color for Domains 1-3 (741 bp, 91-831), and lighter blue for Domains 4 and 5 (480 bp, 832-1311) which are absent from the TrTGM1 construct. The 8-aa FLAG tag with a stop codon (27bp, 1312-1338), immediately after the TGM1 sequence, is labeled in mauve.

Supplementary Figure 2. Expression and Purification of Algal TGM1

A. Mass spectrometry coverage of FLAG-purified algal TGM1. Amino acid sequences highlighted in red are peptides identified from mass spectrometry; blue amino acids are cysteine residues that are alkylated post-reduction; green amino acid sequences are the potential glycosylation sites. The lysine (K) and arginine (R) residues are underlined as potential and identified sites of tryptic cleavage.

B. Anti-FLAG Western blot of FLAG-purified algal TGM1 digested with (+) and without (-) Endo H and with (+) and without (-) PNGase F endoglycosidases. Marker protein molecular weights are indicated.

Supplementary Figure 3. In vitro assays for TGF-β activity.

A. MFB-F11 assay including WT algae protein controls, HEK-expressed and algal-expressed TGM1.

B. Foxp3 induction in $CD4^+$ T cells from a Foxp3-GFP transgenic reporter mouse by HEK-expressed and algal-expressed TGM1 and TrTGM1 in the presence or absence of IL-2 and retinoic acid (RA).

Supplementary Figure 4. Physiological responses to orally administered algal TGM1.

Mice given WT algal proteins, or algal-expressed TGM1 as shown in **Figure 3A** were monitored over 8 days for water intake (**A**), food consumed (**B**), and weight change (**C**).

Supplementary Figure 5. TGM1 administration during DSS-induced colitis.

A. Water intake over 14 days in each experimental group. Expression of RORγt in Tregs from the MLN (**B**) and Peyer's Patches (**C**). UC = untreated control animals.

Supplementary Table 1. Protein yields of secreted algal material. Total protein amount of each sample was quantified by Pierce™ BCA Protein Assay Kit at 562 nm and standardized with 250 µg/mL, 500 µg/mL, 750 µg/mL and 1000 µg/mL bovine serum albumin. Algal TGM1 concentration in precipitated supernatant were quantified by western blot against 150 ng Recombinant Posi-Tag Epitope Tag Protein containing the epitope FLAG tag. Protein band pixel intensities were analyzed by Image Studio Lite.

	**Total protein concentration**	**Volume**	**sTGM concentration in total protein samples**
**Proteins purified from WT supernatant**	**10510 ng/µL**	**250 mL**	**0**
**Protein purified from TGM1 transgenic strain supernatant**	**11850 ng/µL**	**220 mL**	**226 ng/µL**

Supplementary Table 2. Glycosylation site analysis of algal TGM1. A tryptic digest of algal TGM1 was analyzed by mass spectrometry for peptide identification and glycosylation site analysis. Data were analyzed using BioPharma Finder 4.0 software. Glycopeptides with a confidence value of > 80 %, and delta mass of 5 ppm were accepted. Glycosylated residues are highlighted by underlining of asparagine (N) or threonine (T). Cysteine residues alkylated as part of the sample processing are indicated by italics. Of the glycopeptides identified, a nonglycosylated counterpart peptide was only identified for N118. Numbering corresponds to the mature amino acid sequence after cleavage of the 30-aa signal peptide (See **Supplementary Figure 2**).

N or O glycosylation	***Glycosylation site***	***Identification***	***Peptide sequence***	***Glycan***
N	N88	C112-K127	*C*SPLPTNDTVSFEYLK	M5
N	N88	C112-K127	*C*SPLPTNDTVSFEYLK	M5
N	N88	C112-K127	*C*SPLPTNDTVSFEYLK	M4
N	N88	C112-K127	*C*SPLPTNDTVSFEYLK	M4
N	N88	R111-K127	R*C*SPLPTNDTVSFEYLK	M7
N	N88	C112-K127	*C*SPLPTNDTVSFEYLK	M5
N	N88	C112-K127	*C*SPLPTNDTVSFEYLK	M5
N	N88	C112-K127	*C*SPLPTNDTVSFEYLK	None
N	N88	C112-K127	*C*SPLPTNDTVSFEYLK	None
N	N88	C112-K127	*C*SPLPTNDTVSFEYLK	None
N	N107	F136-K147	FNITVHPDASGK	A2G2
N	N107	F136-K147	FNITVHPDASGK	A2G2
N	~N107	L115-K147	LPTNDTVSFEYLKATVNPGIIFNITVHPDASGK	A2G0F
N	N255	P281-T296	PLEANESVHYEYFTMT	A1G1F
N	N255	P281-T296	PLEANESVHYEYFTMT	A1G1F
N	N255	P281-T296	PLEANESVHYEYFTMT	A1G0F
N	N255	P281-T296	PLEANESVHYEYFTMT	A1G0F
N	N255	P281-T294	PLEANESVHYEYFTMT	A2G1B
N	N267	S287-K302	SVHYEYFTMTNETDKK	A2G0B
N	N267	S287-K302	SVHYEYFTMTNETDKK	A2G0B
N	N267	V288-K302	VHYEYFTMTNETDKK	A2G1F
N	~N267	K280-K311	KPLEANESVHYEYFTMTNETDKKKGPPAKVGK	A2G0F
N	~N267	K280-K311	KPLEANESVHYEYFTMTNETDKKKGPPAKVGK	A2G0F
O	~T157	N160-C188	NFPTDSNVQGHIIGM*C*YNAEWQFSSTPT*C*	GalNAc-3G
O	~T157	N160-C188	NFPTDSNVQGHIIGM*C*YNAEWQFSSTPT*C*	GalNAc-3G

Smyth *et al.*, Supplementary Figure 1

ars1 SP

TGM-1 Domains 1-3

TGM-1 Domains 4+5

FLAG tag

```
        10         20         30         40         50
ATGCATGCAC GCAAGATGGG TGCCCTCGCG GTGCTCGCCG TCGCTTGCCT
        60         70         80         90        100
CGCGGCAGTG GCATCGGTTG CGCATGCGGC CGACACCAAA GACGACAGCG
       110        120        130        140        150
GCTGCATGCC CTTCAGCGAC GAGGCCGCCA CCTACAAGTA CGTGGCCAAG
       160        170        180        190        200
GGCCCCAAGA ACATCGAGAT CCCCGCCCAG ATCGACAACA GCGGCATGTA
       210        220        230        240        250
CCCCGACTAC ACCCACGTGA AGCGCTTCTG CAAGGGCCTG CACGGCGAGG
       260        270        280        290        300
ACACCACCGG CTGGTTCGTG GGCATCTGCC TGGCCAGCCA GTGGTACTAC
       310        320        330        340        350
TACGAGGGCG TGCAGGAGTG CGACGACCGC CGCTGCAGCC CCCTGCCCAC
       360        370        380        390        400
CAACGACACC GTGAGCTTCG AGTACCTGAA GGCCACCGTG AACCCCG[illegible]
       410        420        430        440        450
TCATCTTCAA CATCACCGTG CACCCCGACG CCAGCGGCAA GTACCCC[illegible]
       460        470        480        490        500
CTGACCTACA TCAAGCGCAT CTGCAAGAAC TTCCCCACCG ACAGCAAC[illegible]
       510        520        530        540        550
GCAGGGCCAC ATCATCGGCA TGTGCTACAA CGCCGAGTGG CAGTTCAGCA
       560        570        580        590        600
GCACCCCCAC CTGCCCCGCC AGCGGCTGCC CCCCCCTGCC CGACGACGGC
       610        620        630        640        650
ATCGTGTTCT ACGAGTACTA CGGCTACGCC GGCGACCGCC ACACCGTGGG
       660        670        680        690        700
CCCCGTGGTG ACCAAGGACA GCAGCGGCAA CTACCCCAGC CCCACCCACG
       710        720        730        740        750
CCCGCCGCCG CTGCCGCGCC CTGAGCCAGG AGGCCGACCC CGGCGAGTTC
       760        770        780        790        800
GTGGCCATCT GCTACAAGAG CGGCACCACC GGCGAGAGCC ACTGGGAGTA
       810        820        830        840        850
CTACAAGAAC ATCGGCAAGT GCCCCGACCC CCGCTGCAAG CCCCTGGAGG
       860        870        880        890        900
CCAACGAGAG CGTGCACTAC GAGTACTTCA CCATGACCAA CGAGACCGAC
       910        920        930        940        950
AAGAAGAAGG GCCCCCCCGC CAAGGTGGGC AAGAGCGGCA AGTACC[illegible]
       960        970        980        990       1000
GCACACCTGC GTGAAGAAGG TGTGCAGCAA GTGGCCCTAC ACCTGCA[illegible]
      1010       1020       1030       1040       1050
CCGGCGGCCC CATCTTCGGC GAGTGCATCG GCGCCACCTG GAACTT[illegible]
      1060       1070       1080       1090       1100
GCCCTGATGG AGTGCATCAA CGCCCGCGGC TGCAGCAGCG ACGACCTGTT
      1110       1120       1130       1140       1150
CGACAAGCTG GGCTTCGAGA AGGTGATCGT GCGCAAGGGC GAGGGCAGCG
      1160       1170       1180       1190       1200
ACAGCTACAA GGACGACTTC GCCCGCTTCT ACGCCACCGG CAGCAAGGTG
      1210       1220       1230       1240       1250
ATCGCCGAGT GCGGCGGCAA GACCGTGCGC CTGGAGTGCA GCAACGGCGA
      1260       1270       1280       1290       1300
GTGGCACGAG CCCGGCACCA AGACCGTGCA CCGCTGCACC AAGGACGGCA
      1310       1320       1330
TCCGCACCCT GGACTACAAG GACGACGACG ACAAGTAA
```

Smyth *et al.*, Supplementary Figure 2

A

```
□ 1 DDSGCMPFSD EAATYKYVAK GPKNIEIPAQ IDNSGMYPDY THVKRFCKGL
 51 HGEDTTGWFV GICLASQWYY YEGVQECDDR RCSPLPTNDT VSFEYLKATV
101 NPGIIFNITV HPDASGKYPE LTYIKRICKN FPTDSNVQGH IIGMCYNAEW
151 QFSSTPTCPA SGCPPLPDDG IVFYEYYGYA GDRHTVGPVV TKDSSGNYPS
201 PTHARRRCRA LSQEADPGEF VAICYKSGTT GESHWEYYKN IGKCPDPRCK
251 PLEANESVHY EYFTMTNETD KKKGPPAKVG KSGKYPEHTC VKKVCSKWPY
301 TCSTGGPIFG ECIGATWNFT ALMECINARG CSSDDLFDKL GFEKVIVRKG
351 EGSDSYKDDF ARFYATGSKV IAECGGKTVR LECSNGEWHE PGTKTVHRCT
401 KDGIRTL
```

B

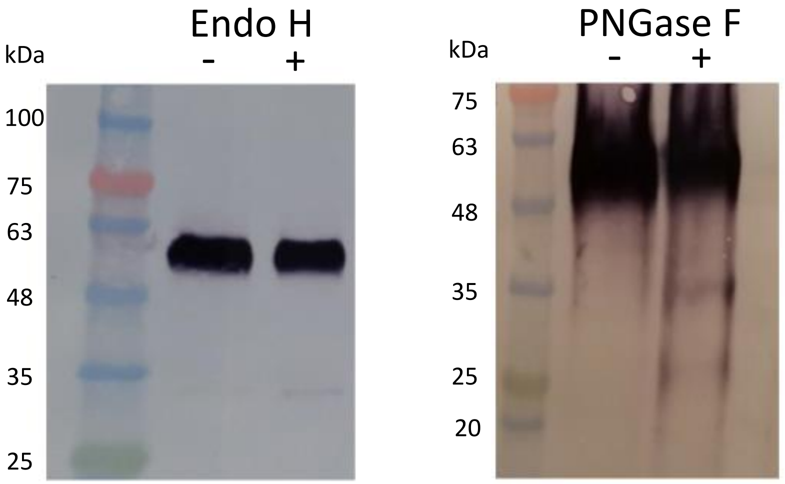

Smyth *et al.*, Supplementary Figure 3

A

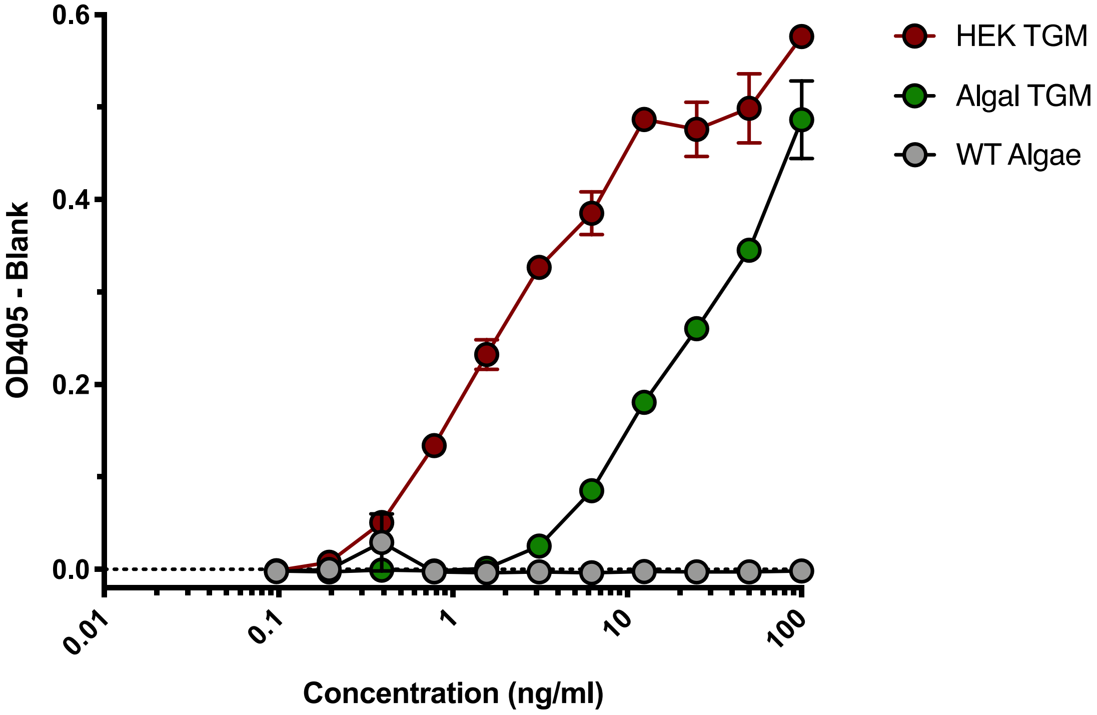

B

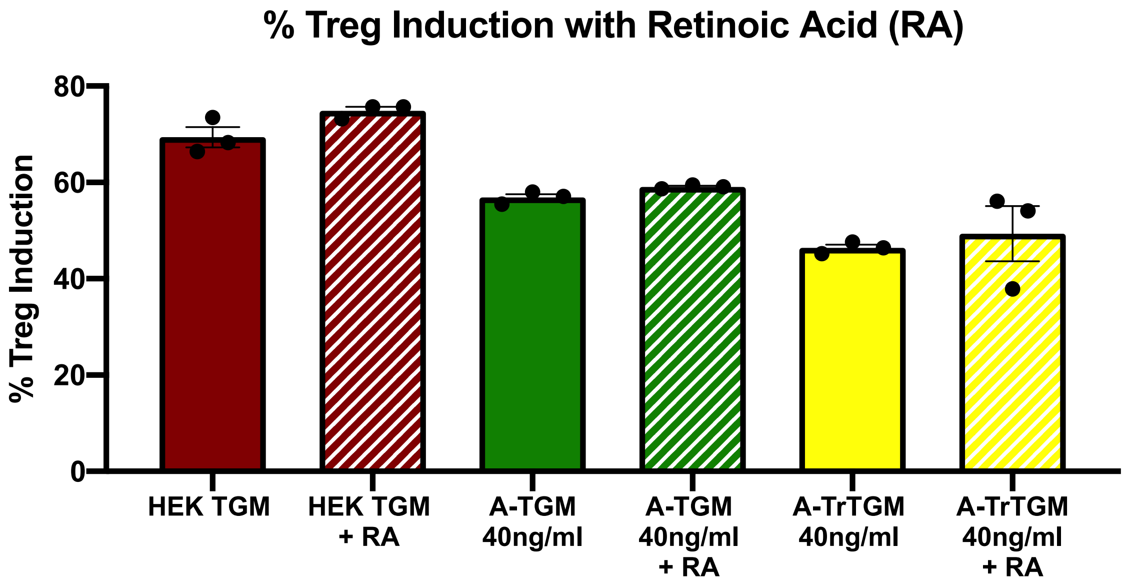

Smyth *et al.*, Supplementary Figure 4

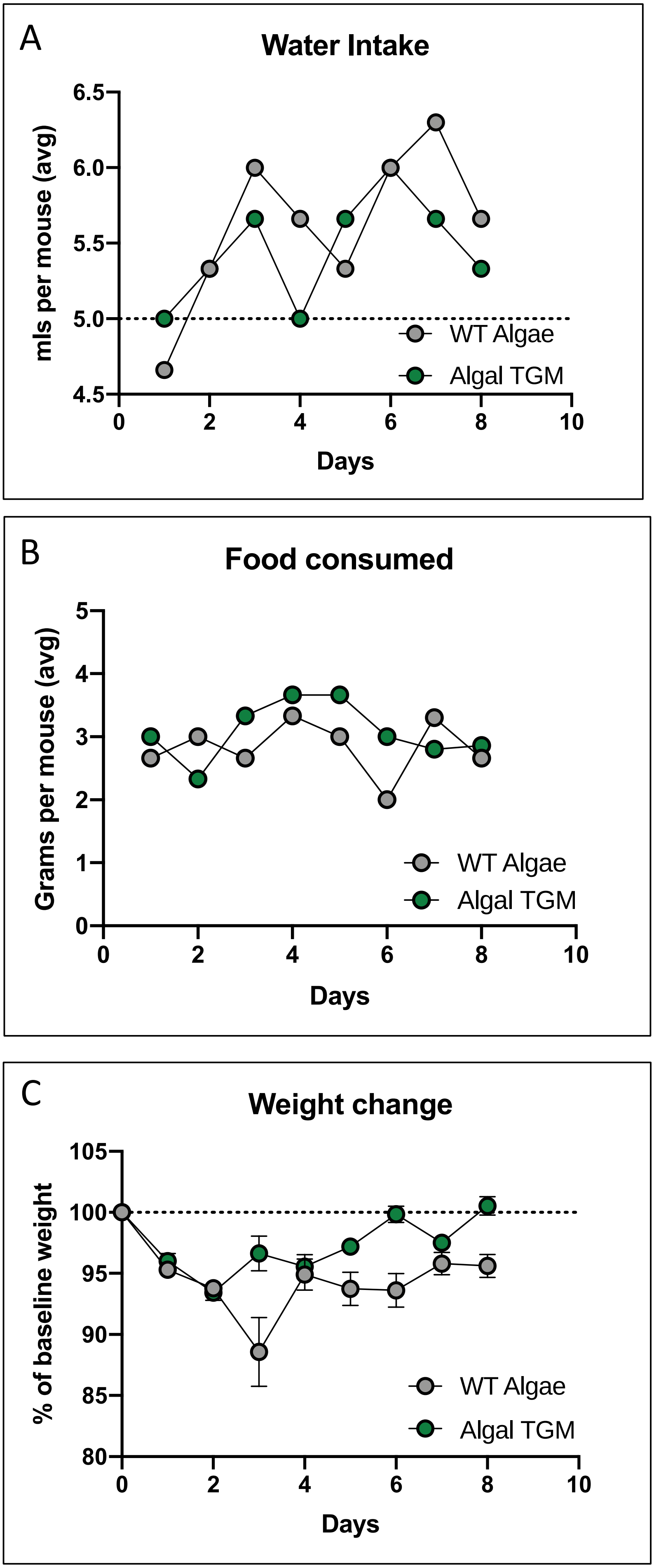

Smyth *et al.*, Supplementary Figure 5

A

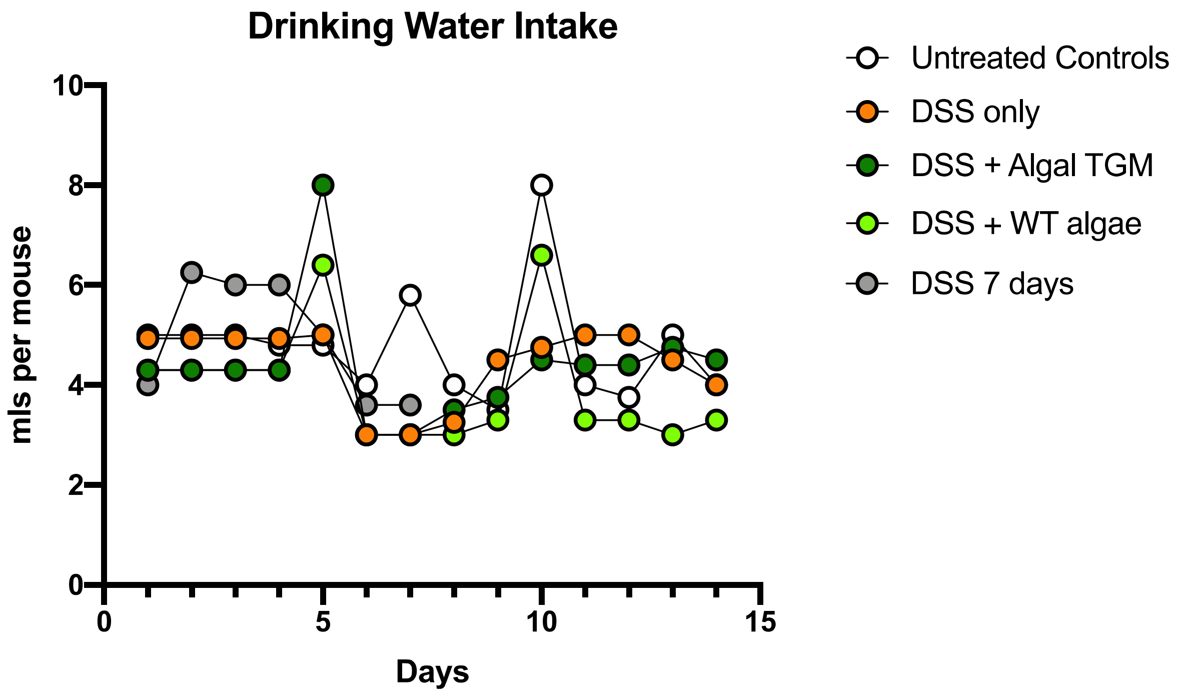

B

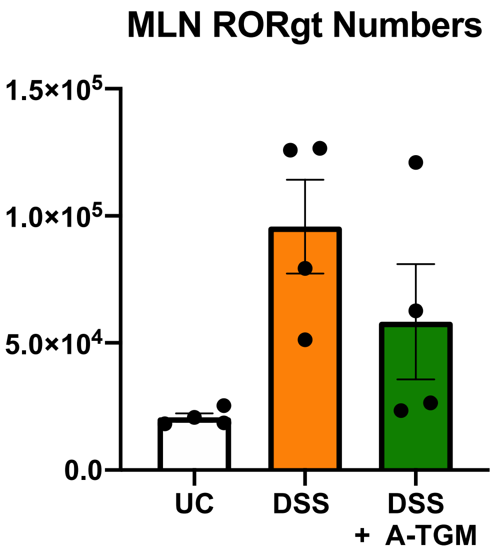

C

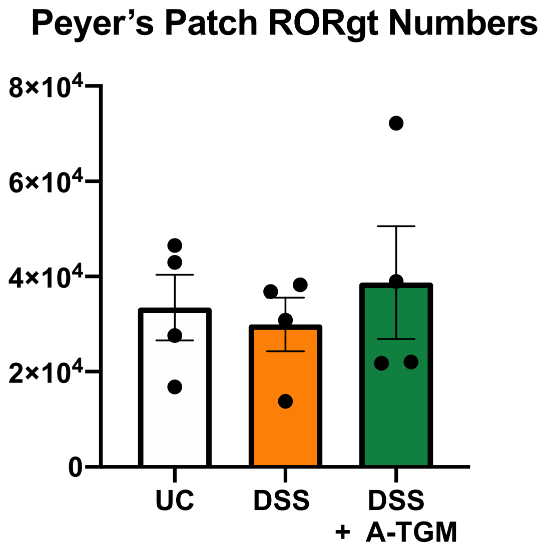

Supplement: Supplementary file 1 — Supplementary material [file mmc1.pdf]
